# Early Outcomes of Bolus Thrombolysis Plus Manual Aspiration Thrombectomy Using Conventional Catheters for Intermediate-High- and High-Risk Acute Pulmonary Embolism

**DOI:** 10.3390/jcm15187157

**Published:** 2026-09-15

**Authors:** Gayoun Min, Wonju Hong, Lyo Min Kwon, Young Soo Do, Hyunseung Nam, Sunghoon Park, Sang-Ho Jo, Hyoung Soo Kim

**Affiliations:** 1 Department of Medicine, College of Medicine, Hallym University, Chuncheon 24252, Republic of Korea; 2 Department of Radiology, Hallym University Sacred Heart Hospital, 22 Gwanpyeong-ro 170beon-gil, Dongan-gu, Anyang 14068, Republic of Korea; 3 Department of Critical Care Medicine, Hallym University Sacred Heart Hospital, Anyang 14068, Republic of Korea; 4 Division of Cardiology, Department of Internal Medicine, Hallym University Sacred Heart Hospital, Anyang 14068, Republic of Korea; 5 Department of Thoracic and Cardiovascular Surgery, Hallym University Sacred Heart Hospital, Anyang 14068, Republic of Korea

**Keywords:** pulmonary embolism, catheters, thrombectomy, thrombolytic therapy, right ventricular dysfunction

## Abstract

**Background:** Dedicated pulmonary embolism (PE) thrombectomy devices are not universally available. We evaluated the early outcomes and safety of catheter-directed bolus thrombolysis plus manual aspiration thrombectomy (BT-MAT) using conventional catheters for intermediate-high- or high-risk acute PE. **Methods:** This retrospective, single-center, historical control study included adults with acute intermediate-high- or high-risk PE involving the main pulmonary artery. Patients who underwent BT-MAT from November 2023 to October 2025 were compared with historical controls who received conventional management such as anticoagulation or systemic thrombolysis before BT-MAT implementation (November 2022 to October 2023). BT-MAT comprised catheter-directed bolus alteplase injection followed by manual aspiration using conventional 7- to 8-Fr catheters or sheaths. **Results:** Twenty-three patients were included (BT-MAT, *n* = 11; historical control, *n* = 12). BT-MAT patients were younger (65.3 ± 16.5 vs. 78.2 ± 11.4 years; *p* = 0.040) and more frequently had bilateral main pulmonary artery involvement (82% vs. 33%; *p* = 0.036). Technical success was achieved in all BT-MAT procedures. Clinical success occurred in 11/11 BT-MAT patients and 10/12 conventional-treatment patients (100.0% vs. 83.3%; *p* = 0.478). No treatment-related complications occurred in the BT- MAT group, whereas two patients in the historical control group developed blood-tinged sputum and hematuria. Estimated right ventricular systolic pressure decreased from 47.1 ± 8.6 to 36.2 ± 10.1 mmHg after BT-MAT. Hospital stay did not differ significantly between groups. **Conclusions:** BT-MAT was technically feasible and showed favorable early clinical outcomes in this small series. Further prospective studies are warranted to evaluate its efficacy and safety.

## 1. Introduction

Acute pulmonary embolism (PE) remains an important cause of morbidity and mortality, with an incidence of approximately 14 per 100,000 population and a mortality rate of approximately 1 per 100,000 population [1]. Because the clinical spectrum of acute PE ranges from low-risk disease to life-threatening hemodynamic collapse, accurate risk stratification is essential for selecting the appropriate treatment strategy. The 2019 European Society of Cardiology guidelines classify patients with acute PE into low-, intermediate-low-, intermediate-high-, and high-risk groups based on clinical status, hemodynamic parameters, right ventricular dysfunction, and cardiac biomarkers [2]. In patients with low- or intermediate-low-risk PE, anticoagulation is recommended as the mainstay of treatment [2]. In contrast, patients with high-risk PE may benefit from reperfusion therapy, including systemic thrombolysis, given the risk of hemodynamic deterioration and early mortality [2].

However, systemic thrombolysis is associated with a substantial risk of bleeding complications, including major bleeding and intracranial hemorrhage, and may be contraindicated or ineffective in some patients [2,3,4]. In patients with intermediate-high-risk PE, the use of systemic thrombolysis remains particularly challenging as the potential benefit of hemodynamic improvement must be balanced against the increased risk of major bleeding [3,4]. Therefore, catheter-directed reperfusion therapy has emerged as an alternative strategy in selected patients, particularly when systemic thrombolysis is contraindicated, has failed, or when rapid thrombus reduction is clinically required [2,5,6,7].

Several catheter-directed approaches have been investigated for the treatment of acute PE. Ultrasound-assisted catheter-directed thrombolysis has demonstrated improvement in right ventricular dysfunction in patients with intermediate-risk PE [5,6]. The PERFECT registry also reported favorable clinical outcomes after catheter-directed mechanical or pharmacomechanical therapy in patients with massive and submassive PE [7]. More recently, dedicated aspiration thrombectomy systems have shown promising results in patients with intermediate-risk PE, including significant reductions in the right ventricular-to-left ventricular diameter ratio and acceptable safety profiles [8,9]. Nevertheless, dedicated PE thrombectomy devices are not universally available, and their use may be limited in resource-limited or non-specialized clinical settings.

To address this practical limitation, we developed and implemented a catheter-based treatment strategy consisting of catheter-directed bolus thrombolytic injection followed by percutaneous aspiration thrombectomy using conventional catheters. In this study, this approach was defined as bolus thrombolysis plus manual aspiration thrombectomy (BT-MAT). This strategy was intended to achieve rapid thrombus reduction while avoiding prolonged thrombolytic infusion and minimizing systemic thrombolytic exposure. Previous reports have suggested that pulmonary artery aspiration thrombectomy using conventional, non-PE-specific catheter devices is technically feasible, although the available evidence remains limited and is largely based on small case series or case reports [10,11,12,13].

Therefore, this study aimed to evaluate the early clinical outcomes and safety of BT-MAT in patients with intermediate-high- or high-risk acute PE involving the main pulmonary artery, and to compare these outcomes with those of a historical control group receiving conventional treatment.

## 2. Materials and Methods

### 2.1. Study Design and Population

This retrospective, single-center, historical control study was approved by the Institutional Review Board of Hallym University Sacred Heart Hospital (IRB No. 2026-01-001-002). The requirement for informed consent was waived. The study evaluated the clinical outcomes and safety of BT-MAT in patients with acute PE involving the main pulmonary artery. Patients were divided into two groups according to treatment strategy and study period. The BT-MAT group included consecutive patients with acute PE who underwent BT-MAT between November 2023 and October 2025. The historical control group consisted of patients newly diagnosed with acute PE between November 2022 and October 2023, who were managed with conventional treatment before the implementation of BT-MAT at our institution. Conventional treatment was defined as anticoagulation-based management according to institutional practice, with systemic thrombolysis administered when clinically indicated at the treating physician’s discretion.

Patients were eligible if they met all of the following criteria: (1) newly diagnosed acute PE involving the left or right main pulmonary artery on computed tomography (CT) pulmonary angiography; (2) intermediate-high- or high-risk PE according to the 2019 European Society of Cardiology (ESC) guidelines [2]; and (3) hospitalization and treatment at our institution during the predefined study period. Patients meeting any of the following criteria were excluded: (1) age younger than 20 years; (2) low-risk or intermediate-low-risk PE; (3) absence of main pulmonary artery involvement; or (4) contraindications to thrombolytic therapy.

### 2.2. BT-MAT Procedure

BT-MAT was considered in patients with right ventricular (RV) dysfunction and a large thrombus burden involving the main pulmonary arteries whose symptoms persisted or deteriorated despite anticoagulation. The decision to perform BT-MAT was made through discussion within a multidisciplinary PE team comprising specialists in interventional radiology, cardiology, critical care medicine, and thoracic surgery.

BT-MAT was performed in an angiography suite by two board-certified interventional radiologists with 8 and over 30 years of clinical experience, respectively. Under ultrasound guidance, venous access was obtained through the right common femoral vein. A 10-Fr vascular sheath was inserted, and a guiding sheath or guiding catheter, usually an 8-Fr Ansel guiding sheath (Merit Medical Systems, Inc., South Jourdan, UT, USA), was advanced into the main pulmonary artery or the affected pulmonary artery under fluoroscopic guidance. Initial pulmonary angiography was performed to confirm thrombus location and burden. Tissue plasminogen activator (alteplase; Actilyse, Boehringer Ingelheim, Ingelheim am Rhein, Germany) was administered as a catheter-directed bolus through a catheter positioned within the affected pulmonary artery. The total dose was determined by the operator according to thrombus burden, hemodynamic status, bleeding risk, and overall clinical condition. Up to 15 mg was administered for unilateral main pulmonary artery involvement and up to a total of 25 mg for bilateral main pulmonary artery involvement, without exceeding the recommended weight-based maximum dose for systemic thrombolysis. A short dwell period of approximately 30 min was allowed before aspiration when feasible. After bolus thrombolytic injection, aspiration thrombectomy was performed using conventional aspiration-compatible catheters, including a 7-Fr ENVOY guiding catheter (Codman & Shurtleff Inc., Raynham, MA, USA) or an 8-Fr FUBUKI guiding catheter (Asahi Intecc, Aichi, Japan). Manual aspiration was performed using a syringe while the catheter tip was positioned at or near the thrombotic segment. Aspiration was repeated until angiographic improvement was observed and no further thrombus could be aspirated. Dedicated PE-specific aspiration thrombectomy systems, such as the FlowTriever or Indigo systems, were not used. Procedural details, including access route, thrombolytic dose, procedure time, use of extracorporeal membrane oxygenation, and periprocedural complications, were recorded.

### 2.3. Data Collection

Clinical, laboratory, imaging, procedural, and outcome data were retrospectively collected from EMRs, radiology reports, angiographic images, and echocardiographic reports. Baseline demographic and clinical variables included age, sex, body mass index, comorbidities, history of prior deep vein thrombosis or PE, Eastern Cooperative Oncology Group (ECOG) performance status [14], and Pulmonary Embolism Severity Index (PESI) score [15]. Clinical presentation data included initial symptoms, oxygen saturation, oxygen demand, cardiac arrest, syncope, altered mentality, persistent hypotension, vasopressor requirement and vital signs, including systolic and diastolic blood pressure, heart rate, respiratory rate, and body temperature. Laboratory variables included D-dimer, troponin T, brain natriuretic peptide, and lactate levels. Imaging variables included thrombus distribution, unilateral or bilateral main pulmonary artery involvement, presence and level of concomitant deep vein thrombosis, and right ventricular strain on imaging. Echocardiographic data, when available, included right ventricular dysfunction, right ventricular S′ velocity, tricuspid annular plane systolic excursion, fractional area change, estimated right ventricular systolic pressure (eRVSP), and pulmonary hypertension severity. Pulmonary hypertension severity was categorized according to eRVSP as follows: normal, ≤34 mmHg; mild, 35–49 mmHg; moderate, 50–69 mmHg; and severe, ≥70 mmHg [16].

### 2.4. Outcome Measures

The primary outcome was clinical success. Clinical success was defined as fulfillment of all of the following criteria during hospitalization: (1) hemodynamic stabilization, defined as maintenance of systolic blood pressure ≥ 90 mmHg without escalation of vasopressor support; (2) improvement or resolution of hypoxia, defined as a reduction in oxygen demand or successful weaning from supplemental oxygen; and (3) survival to discharge without PE-related in-hospital mortality. Patients who were transferred to long-term care hospitals with persistent oxygen demand were not considered to have achieved clinical success. Secondary outcomes included technical success, echocardiographic hemodynamic changes, and hospital stay. Technical success was assessed only in the BT-MAT group and was defined as successful catheter navigation to the target pulmonary artery, completion of catheter-directed bolus thrombolytic injection, and completion of aspiration thrombectomy according to the planned procedural strategy. Echocardiographic outcomes included changes in estimated right ventricular systolic pressure (eRVSP), right ventricular functional parameters, and pulmonary hypertension severity before and after treatment. Hospital utilization outcomes included intensive care unit stay, general ward stay, and total hospital stay. Safety outcomes included treatment-related complications during hospitalization, including major or minor bleeding, access-site complications, bloody sputum, hematuria, neurologic complications, infection, and other clinically relevant adverse events.

### 2.5. Follow-Up Assessment

Clinical follow-up was performed during hospitalization until discharge or transfer. Post-treatment oxygen demand, vital signs, symptoms, complications, and survival status were extracted from EMRs. Follow-up echocardiography and CT were performed according to the decision of the treating physician and clinical necessity. Because this was a retrospective study, the timing and availability of follow-up imaging were not standardized.

### 2.6. Statistical Analysis

Continuous variables were assessed for normality using the Shapiro–Wilk test. Normally distributed variables were presented as mean ± standard deviation and compared using the independent-samples *t*-test. Non-normally distributed variables were presented as median with interquartile range or range and compared using the Mann–Whitney U test. For paired pre- and post-treatment comparisons, the paired t-test or Wilcoxon signed-rank test was used as appropriate. Categorical variables were presented as numbers and percentages. Between-group comparisons of categorical variables were performed using Fisher’s exact test. For paired categorical variables, McNemar test was used when applicable. Given the small sample size and exploratory nature of this study, multivariable regression analysis was not performed. For the principal binary outcomes, absolute risk differences were calculated as the BT-MAT group minus the historical control group and reported with exact unconditional 95% confidence intervals based on the standardized score statistic. Statistical analyses were conducted using Jamovi software, version 2.3.21 (The jamovi project, Sydney, Austrailia). A two-sided *p*-value < 0.05 was considered statistically significant.

## 3. Results

**Figure 1:**
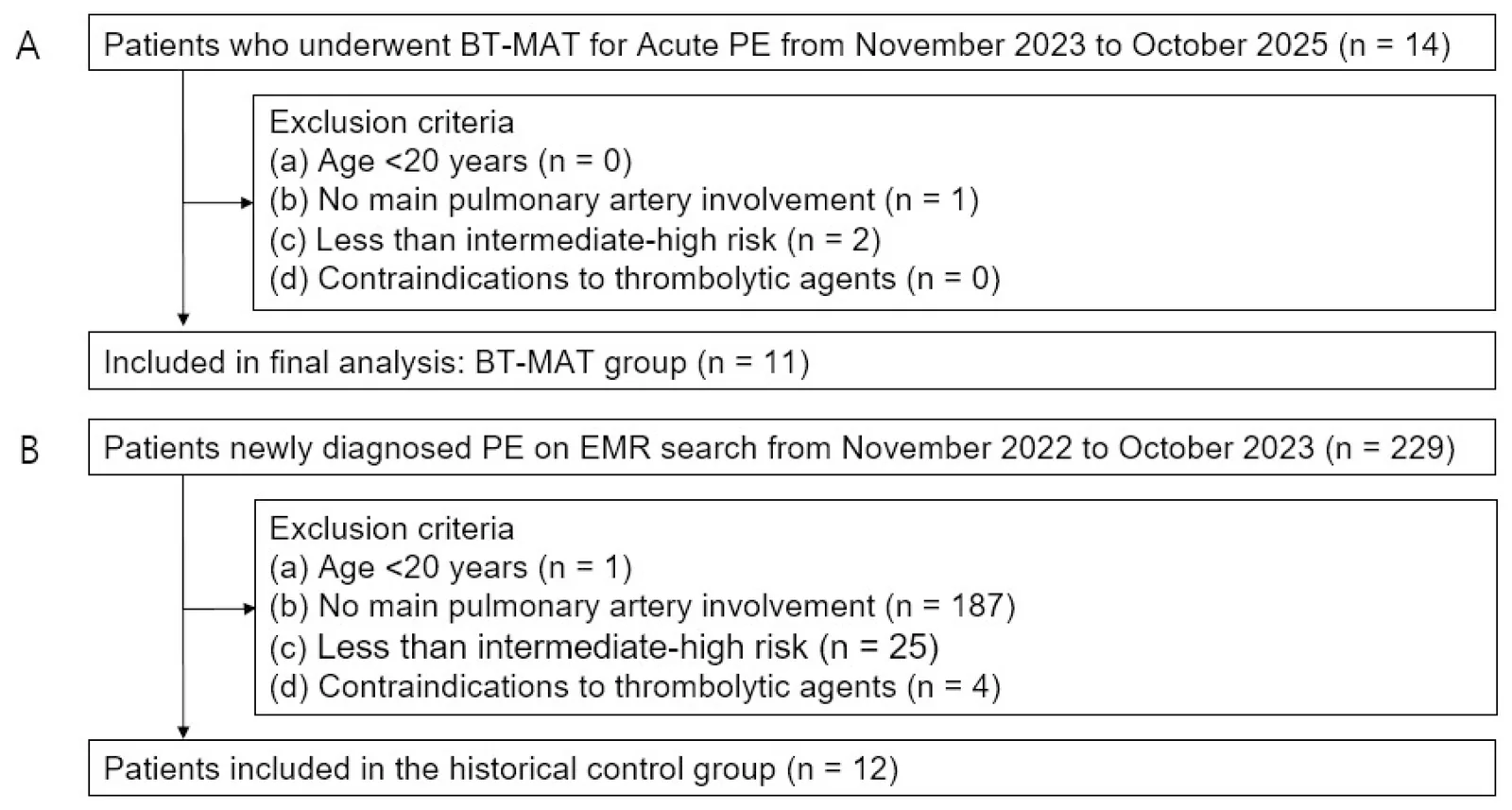
Patient selection and study flowchart. (**A**) Patients with acute pulmonary embolism (PE) who underwent bolus thrombolysis followed by manual aspiration thrombectomy (BT-MAT). (**B**) Historical control group comprising patients newly diagnosed with PE on electronic medical record (EMR) search. Abbreviations: PE, pulmonary embolism; BT-MAT, bolus thrombolysis-manual aspiration thrombectomy; EMR, electronic medical record.

During the BT-MAT study period, 14 patients underwent BT-MAT for acute PE. Three patients were excluded: one because of the absence of main pulmonary artery involvement and two because they had low- or intermediate-low-risk PE. During the historical control period, 229 patients newly diagnosed with acute PE were identified through EMR screening. Of these, 217 were excluded according to the predefined exclusion criteria. Overall, 23 patients were included in the final analysis: 11 in the BT-MAT group and 12 in the historical control group (Figure 1).

Baseline demographic and clinical characteristics are summarized in Table 1. Patients in the BT-MAT group were significantly younger than those in the historical control group (65.3 ± 16.5 years vs. 78.2 ± 11.4 years, *p* = 0.040), and the proportion of female patients was lower (3/11, 27% vs. 10/12, 83%; *p* = 0.012). The BT-MAT group had greater radiologic PE burden, with more frequent bilateral main pulmonary artery involvement (9/11, 82% vs. 4/12, 33%; *p* = 0.036) and a higher RV/left ventricular (LV) ratio (1.53 [1.26–1.81] vs. 1.22 [0.88–1.35]; *p* = 0.038). Although the overall PESI score did not differ significantly between groups, PESI class distribution differed significantly (*p* = 0.017), with a higher proportion of patients classified as PESI class V in the BT-MAT group (6/11, 55% vs. 4/12, 33%). ESC severity risk category did not differ significantly between groups.

Treatment characteristics are shown in Supplementary Table S1. Extracorporeal membrane oxygenation support was used in one patient in each group, and anticoagulation was administered in all patients. In the BT-MAT group, the median time from diagnosis to procedure was 12 h (interquartile range, 9–33 h), and the mean procedure time was 113.5 ± 30.9 min. The mean tissue plasminogen activator dose used for bolus thrombolysis was 13.9 ± 5.9 mg (range, 6–24 mg). In the historical control group, systemic thrombolysis was administered in one patient.

**Figure 2:**
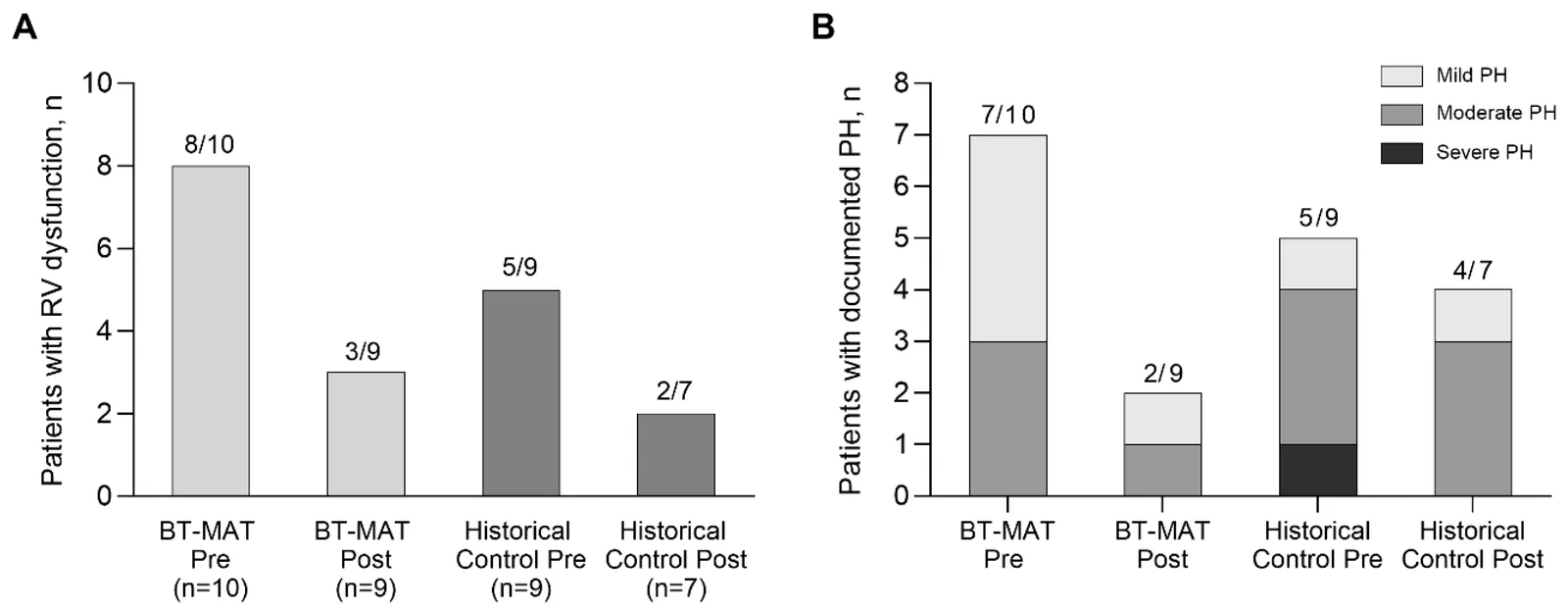
Echocardiographic changes after treatment in the BT-MAT and historical control groups. (**A**) Proportion of patients with right ventricular (RV) dysfunction before and after treatment in the BT-MAT and historical control groups. RV dysfunction decreased after treatment in both groups, with a greater numerical reduction observed in the BT-MAT group. Values above bars indicate the number of patients with RV dysfunction relative to the number of evaluable patients. (**B**) Distribution of documented pulmonary hypertension (PH) severity before and after treatment in the BT-MAT and historical control groups. PH severity was classified according to estimated right ventricular systolic pressure (eRVSP) as mild (35–49 mmHg), moderate (50–69 mmHg), and severe (≥70 mmHg). The BT-MAT group showed a numerical shift toward lower PH severity after treatment. Values above bars indicate the number of patients with documented PH relative to the number of evaluable patients. Abbreviations: BT-MAT, bolus thrombolysis plus manual aspiration thrombectomy; PH, pulmonary hypertension; RV, right ventricular.

Echocardiography was performed before and after treatment in 10 of 11 and 9 of 11 patients in the BT-MAT group, respectively, and in 9 of 12 and 7 of 12 patients in the historical control group, respectively (Supplementary Table S2). The median interval to follow-up echocardiography was 7 days (IQR, 3–17 days) in the BT-MAT group and 31 days (IQR, 18–97 days) in the conventional treatment group. In the BT-MAT group, right ventricular dysfunction decreased from 8 of 10 to 3 of 9 evaluable patients, whereas it decreased from 5 of 9 to 2 of 7 evaluable patients in the historical control group (Figure 2). Documented pulmonary hypertension decreased from 7 of 10 to 2 of 9 evaluable patients in the BT-MAT group, whereas it remained similar in the historical control group, from 5 of 9 to 4 of 7 evaluable patients. Pulmonary hypertension status and severity also showed a numerical shift toward lower severity after BT-MAT. Among patients with documented pulmonary hypertension, eRVSP decreased from 47.1 ± 8.6 to 36.2 ± 10.1 mmHg in the BT-MAT group and from 60.8 ± 14.7 to 46.8 ± 16.4 mmHg in the historical control group. None of these within-group changes or between-group comparisons of change reached statistical significance.

Clinical outcomes and hospital stay are summarized in Supplementary Table S3. Clinical success was achieved in all patients in the BT-MAT group and in 10 of 12 patients in the historical control group (100.0% vs. 83.3%; absolute risk difference, 16.7 percentage points; exact 95% CI, −14.8 to 48.4; *p* = 0.478). Technical success was achieved in all BT-MAT procedures (11/11, 100.0%).

**Figure 3:**
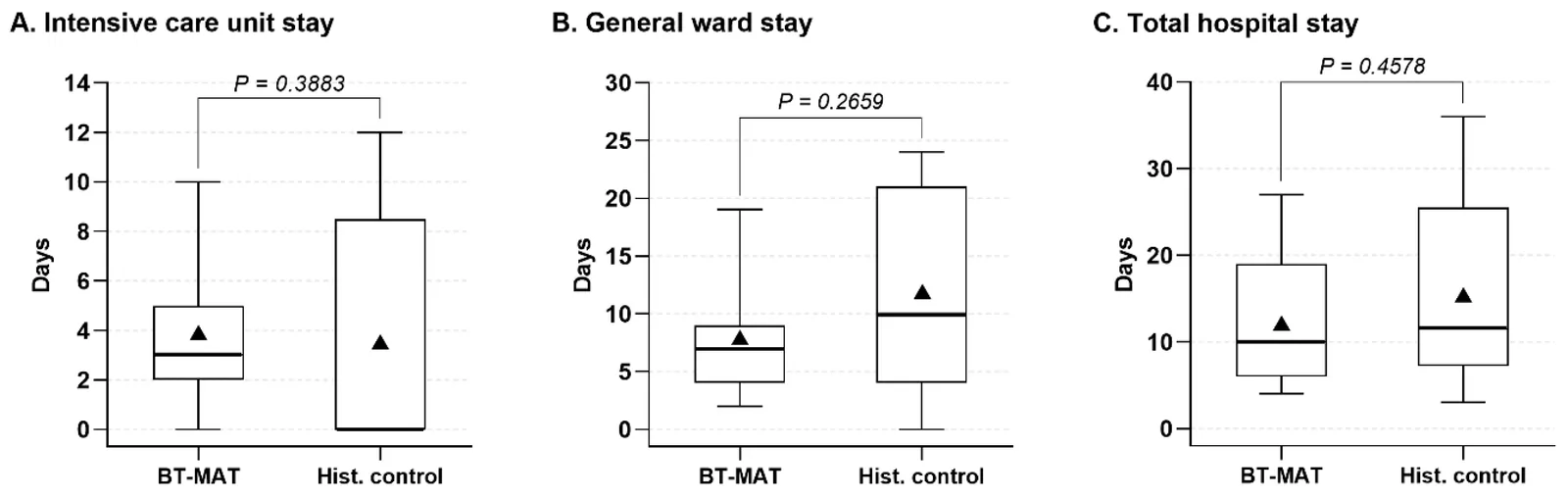
Comparison of clinical outcomes between the BT-MAT and conventional treatment groups. (**A**) Duration of intensive care unit (ICU) stay, (**B**) Duration of general ward stay, and (**C**) Total hospital stay. Data are presented as median and interquartile range (IQR), as well as mean ± standard deviation (SD). (**A**) ICU stay: 3.0 [2.5–4.5] days (mean: 3.7 ± 3.0) in the BT-MAT group vs. 0.0 [0.0–5.5] days (mean: 3.4 ± 5.0) in the conventional group (*p* = 0.3883). (**B**) General ward stay: 7.0 [4.5–8.5] days (mean: 7.9 ± 5.4) in the BT-MAT group vs. 10.0 [6.0–19.0] days (mean: 11.5 ± 8.7) in the conventional group (*p* = 0.2659). (**C**) Total hospital stay: 10.0 [6.5–15.0] days (mean: 11.6 ± 7.6) in the BT-MAT group vs. 11.5 [7.8–20.5] days (mean: 14.9 ± 11.2) in the conventional group (*p* = 0.4578). Individual data points are omitted for clarity, and solid black triangles indicate the mean values for each group. Statistical significance was assessed using the Mann–Whitney U test, and *p*-values are indicated above each plot. Abbreviations: BT-MAT, bolus thrombolysis plus manual aspiration thrombectomy.

No treatment-related complication was observed in the BT-MAT group, whereas two complications occurred in the historical control group (0% vs. 16.7%; absolute risk difference, −16.7 percentage points; exact 95% CI, −48.4 to 14.8; *p* = 0.478): one patient developed bloody sputum with pneumonia and one developed transient hematuria, which was classified as minor bleeding. Regarding hospital stay outcomes, ICU stay showed wide variability, particularly in the historical control group, and did not differ significantly between groups: median, 3.0 days (IQR, 2.5–4.5) vs. 0.0 days (IQR, 0–5.5); mean, 3.7 ± 3.0 vs. 3.4 ± 5.0 days; *p* = 0.389. General ward stay and total hospital stay were numerically shorter in the BT-MAT group than in the historical control group: general ward stay, median 7.0 days (IQR, 4.5–8.5) vs. 10.0 days (IQR, 6.0–19.0), and mean 7.9 ± 5.4 vs. 11.5 ± 8.7 days; total hospital stay, median 10.0 days (IQR, 6.5–15.0) vs. 11.5 days (IQR, 7.8–20.5), and mean 11.6 ± 7.6 vs. 14.9 ± 11.2 days. These differences were not statistically significant (Figure 3).

## 4. Discussion

In this retrospective historical control study, BT-MAT was technically feasible and showed favorable early clinical outcomes in patients with intermediate-high- or high-risk acute PE involving the main pulmonary artery. Technical and clinical success were achieved in all patients in the BT-MAT group, and no treatment-related complications were observed. In the historical control group, clinical success was achieved in 10 of 12 patients, and two complications occurred: bloody sputum with pneumonia, and hematuria. However, given the small sample size and the heterogeneity in bleeding risk according to individual patient characteristics, the safety profile of BT-MAT should be further evaluated in larger prospective studies.

Interpretation of between-group comparisons requires caution because of the nonrandomized study design and differences in baseline characteristics. Although patients in the BT-MAT group were younger, they also tended to have greater radiologic PE burden and a higher proportion of PESI class V patients, suggesting greater clinical severity. Given the small sample size and these substantial baseline imbalances, the present historical comparison should be regarded as exploratory; the absence of statistically significant between-group differences cannot be interpreted as evidence of comparable efficacy or safety. Hospital stay did not differ significantly, although this finding should be interpreted cautiously because the historical control group encompassed a broader range of clinical trajectories and management strategies, whereas patients selected for BT-MAT generally underwent a more active reperfusion approach. Therefore, the present findings should be considered preliminary and primarily support the technical feasibility of BT-MAT.

Current guidelines recommend risk-adapted treatment for acute PE [2]. Systemic thrombolysis remains the standard reperfusion therapy for high-risk PE, whereas surgical embolectomy or catheter-directed treatment may be considered when thrombolysis is contraindicated, has failed, or when rapid hemodynamic deterioration is expected and local expertise is available [2]. Nevertheless, systemic thrombolysis carries a substantial risk of bleeding complications. Both the PEITHO trial and a subsequent meta-analysis demonstrated that thrombolytic therapy, while reducing hemodynamic decompensation, was associated with significantly increased risks of major bleeding, stroke, and intracranial hemorrhage [3,4]. These limitations have motivated the development of catheter-based reperfusion strategies that reduce systemic thrombolytic exposure.

Several catheter-directed therapies have shown favorable outcomes in acute PE. Ultrasound-assisted catheter-directed thrombolysis improved right ventricular dysfunction in the ULTIMA trial [5], and the SEATTLE II study demonstrated reductions in right ventricular dilation, pulmonary hypertension, and thrombus burden after ultrasound-facilitated low-dose fibrinolysis [6]. The PERFECT registry also reported high clinical success rates without major procedure-related complications or major hemorrhage [7]. More recently, dedicated aspiration thrombectomy systems such as FlowTriever and Indigo demonstrated significant reductions in RV/LV ratio with acceptable safety profiles in the FLARE and EXTRACT-PE studies [8,9]. However, many of these catheter-directed approaches rely on specialized thrombolysis catheters or dedicated thrombectomy devices, which may not be universally available. In such settings, catheter-based reperfusion using conventional devices may need to be considered.

In this context, BT-MAT was designed as a pharmacomechanical reperfusion strategy using conventional catheter devices. Alteplase was administered as a catheter-directed bolus into the thrombus-containing pulmonary arterial segment, followed by immediate mechanical thrombus reduction, with the intent of maximizing local thrombolytic effect while limiting prolonged systemic exposure. The pharmacokinetic profile of alteplase provides a pharmacologic rationale for this approach, as its initial plasma half-life is less than 5 min [17]. Direct bolus delivery into a pulmonary arterial segment with markedly reduced flow may allow high local drug concentration while limiting the duration of systemic exposure, and subsequent aspiration thrombectomy may further reduce residual thrombus burden.

Evidence supporting catheter-based reperfusion using conventional devices remains limited but encouraging. Tajima et al. reported manual aspiration thrombectomy using a standard 8-Fr percutaneous transluminal coronary angioplasty guiding catheter in patients with acute massive PE and hemodynamic impairment [10]. The same group subsequently described a hybrid approach combining mechanical fragmentation with a modified rotating pigtail catheter, local fibrinolysis, and manual aspiration [11]. Ruzsa et al. reported catheter-directed thrombolysis with bolus administration followed by maintenance infusion and delayed thrombectomy when needed [12], whereas Tomioka et al. described catheter-directed treatment using conventional catheter devices without dedicated PE thrombectomy systems [13]. However, these studies were mostly small, noncomparative experiences. By evaluating BT-MAT against a historical control group, the present study extends the existing literature by providing early comparative outcome data on a conventional-device-based reperfusion strategy.

In this small cohort, echocardiographic findings showed favorable numerical trends after BT-MAT, but none of the changes reached statistical significance. The proportion of patients with documented pulmonary hypertension decreased after BT-MAT, and pulmonary hypertension severity shifted numerically toward lower severity. Among patients with documented pulmonary hypertension, eRVSP also decreased numerically. Although these findings are insufficient to demonstrate a definitive hemodynamic benefit, they may indicate a potential signal of early pulmonary pressure unloading after BT-MAT. Further studies with larger sample sizes and standardized echocardiographic follow-up are needed to clarify this effect.

Beyond these procedure-specific and echocardiographic findings, early prognosis in acute PE is also influenced by broader patient-specific factors. Renal dysfunction has been shown to provide additional prognostic value for 30-day mortality beyond conventional risk stratification [18]. An elevated neutrophil-to-lymphocyte ratio (>7.0) has also been associated with increased 30-day mortality in acute PE [19]. In addition, real-world registry data have suggested an association between baseline statin use and lower 30-day mortality in patients with acute symptomatic PE [20]. These findings emphasize the importance of considering patient-specific prognostic factors in addition to PE severity when interpreting early clinical outcomes.

This study has several limitations. First, it was retrospective and single-center in design. Second, the historical control group was nonconcurrent, and treatment decisions were influenced by advanced age, concerns about bleeding from systemic thrombolysis, and perioperative mortality from open surgical embolectomy, introducing potential temporal and selection bias. Third, the small sample size and nonrandomized design limited statistical power, precluded robust multivariable adjustment, and may have resulted in residual confounding; moreover, the absence of treatment-related complications in the BT-MAT group should be interpreted cautiously because the cohort was too small to establish procedural safety. Fourth, follow-up echocardiography was not standardized, and several echocardiographic parameters had missing post-treatment data, particularly in the conventional treatment group. Accordingly, these preliminary findings warrant validation in larger prospective studies.

## 5. Conclusions

In conclusion, BT-MAT was technically feasible and showed favorable early clinical outcomes in selected patients with intermediate-high- or high-risk acute PE involving the main pulmonary artery. However, larger prospective studies are needed to confirm its safety and efficacy.

## Figures and Tables

**Table 1:** Demographics.

Variable	BT-MAT Group (*n* = 11)	Historical Control Group (*n* = 12)	*p*-Value
Baseline characteristics			
Age, years	65.3 ± 16.5	78.2 ± 11.4	0.040
Sex, *n* (%)			0.012
Male	8 (73)	2 (17)	
Female	3 (27)	10 (83)	
Body mass index, kg/m^2^	26.4 ± 3.9	24.0 ± 3.9	0.163
ECOG performance status	2.7 ± 1.0	2.8 ± 1.1	0.814
Comorbidities			
Prior DVT or PE, *n* (%)	2 (18)	0 (0)	0.217
Pulmonary disease, *n* (%)	2 (18)	1 (8)	0.590
Cardiovascular disease, *n* (%)	9 (82)	10 (83)	>0.999
Malignancy, *n* (%)	2 (18)	6 (50)	0.193
Other diseases, *n* (%)	10 (91)	9 (75)	0.590
Vital status			
Initial saturation, %	89.4 ± 5.9	94.2 ± 5.7	0.061
O_2_ demand, *n* (%)	8 (73)	7 (58)	0.667
Cardiac arrest, *n* (%)	2 (18)	1 (8)	0.590
Shock, *n* (%)	3 (27)	4 (33)	>0.999
Persistent hypotension, *n* (%)	1 (9)	0 (0)	0.478
Heart rate, beats/min	103.4 ± 26.4	95.1 ± 22.7	0.428
Respiratory rate, breaths/min	20.0 ± 3.2	19.0 ± 5.0	0.581
Body temperature, °C	37.0 ± 0.8	36.7 ± 1.3	0.487
Laboratory finding			
Elevated D-dimer, *n* (%) *	11 (100)	12 (100)	>0.999
Elevated Troponin T, *n* (%) ^†^	10 (91)	10 (83)	>0.999
BNP, pg/mL	186.6 (15.9–9519.7)	117.9 (22.0–908.3)	0.872
Lactate, mmol/L	2.8 ± 2.6	2.8 ± 2.5	0.972
Radiologic findings			
PE burden			0.036
Bilateral main	9 (82)	4 (33)	
Unilateral main	2 (18)	8 (67)	
Concomitant DVT, *n* (%)	7 (64)	8 (67)	>0.999
DVT—Iliac	0 (0)	2 (17)	0.478
DVT—Femoral	6 (55)	4 (33)	0.414
DVT—Below knee	1 (9)	2 (17)	>0.999
RV strain, *n* (%)	5 (45)	8 (67)	0.414
RV/LV ratio	1.53 [1.26–1.81]	1.22 [0.88–1.35]	0.038
RV/LV ratio ≥ 1.0, *n* (%)	9 (82)	7 (58)	0.371
PESI score	133 (79–194)	110 (79–240)	0.853
PESI class			0.017
Class II	4 (36)	0 (0)	
Class III	1 (9)	5 (42)	
Class IV	0 (0)	3 (25)	
Class V	6 (55)	4 (33)	
Severity risk			0.684
Intermediate-high	5 (45)	7 (58)	
High	6 (55)	5 (42)	

Note. Data are presented as mean ± standard deviation, median (range), median [interquartile range] or number (%). *p*-values were calculated using the independent-samples *t*-test, Mann–Whitney U test, or Fisher’s exact test as appropriate. * Elevated D-dimer was defined as ≥0.5 µg/mL. ^†^ Elevated troponin T was defined as ≥14 ng/L. The one patient without elevated troponin T in the BT-MAT group had cardiac arrest, while both patients without elevated troponin T in the historical control group presented with shock. Abbreviations: BNP, brain natriuretic peptide; BT-MAT, bolus thrombolysis plus manual aspiration thrombectomy; DVT, deep vein thrombosis; ECOG, Eastern Cooperative Oncology Group; PE, pulmonary embolism; PESI, Pulmonary Embolism Severity Index; RV/LV, right ventricular/left ventricular.

## Data Availability

The data that support the findings of this study are available from the corresponding author upon reasonable request.

## Supplementary Materials

The following supporting information can be downloaded at https://www.mdpi.com/article/10.3390/jcm15187157/s1: Supplementary Table S1. Treatment characteristics; Supplementary Table S2. Hemodynamic data; Supplementary Table S3. Outcomes.
