# Supplementary material for: Early Outcomes of Bolus Thrombolysis Plus Manual Aspiration Thrombectomy Using Conventional Catheters for Intermediate-High- and High-Risk Acute Pulmonary Embolism
Source: J Clin Med. 2026 Sep 15;15(18):7157. doi: 10.3390/jcm15187157 (PMC13607517; doi:10.3390/jcm15187157)
Supplement: Supplementary file 1 [file jcm-15-07157-s001.zip › jcm-4528311-supplementary.pdf]

Supplementary Table S1. Treatment characteristics

Variable	BT-MAT group (n = 11)	Historical control group (n = 12)
ECMO, n (%)	1 (9)	1 (8)
Systemic thrombolysis, n (%)	NA	1 (8)
Time from diagnosis to procedure, hr	12 (9–33)	NA
Procedure time, min	113.5 ± 30.9	NA
tPA dose, mg	13.9 ± 5.9	NA*
Anticoagulation, n (%)	11 (100)	12 (100)
Apixaban	5 (46)	3 (25)
Rivaroxaban	4 (36)	2 (17)
Edoxaban	1 (9)	2 (17)
Warfarin	1 (9)	0 (0)
Enoxaparin (low-molecular-weight heparin)	0 (0)	5 (42)

Note. Data are presented as mean ± standard deviation, median (range), or number (%).
*One patient in the conventional treatment group received systemic thrombolysis with a total tPA dose of 47 mg (7 mg bolus followed by 40 mg over 2 hours).

Heparin-based anticoagulation in this table is represented by enoxaparin, a low-molecular-weight heparin.
Abbreviations: BT-MAT, bolus thrombolysis plus manual aspiration thrombectomy; ECMO, extracorporeal membrane oxygenation; NA, not applicable; tPA, tissue plasminogen activator.

Supplementary Table S2. Hemodynamic data

Variable	BT-MAT group Pre	BT-MAT group Post	BT-MAT group Within-group P	Historical control group Pre	Historical control group Post	Historical control group Within-group P	Between-group P-value Pre	Between-group P-value Post	Between-group P-value Change
Echocardiography performed, n/N (%)	10/11 (91)	9/11 (82)		9/12 (75)	7/12 (58)		0.590	0.371	
**RV dysfunction**									
Overall presence, n/evaluable (%)	8/10 (80)	3/9 (33)	0.125	5/9 (56)	2/7 (29)		0.350	1.000	0.454
RV S′, cm/s	9.9 ± 3.1	13.4 ± 3.2	0.094	12.5 ± 3.7	13.8 ± 4.1		0.226	1.000	0.386
TAPSE, mm	14.8 ± 3.5	22.0 ± 1.7	0.125	18.3 ± 2.2	20.5 ± 5.8		0.105	0.317	0.140
FAC, %	26.9 ± 9.1	33.6 ± 10.2	0.125	23.8 ± 10.5	32.3 ± 14.6		0.609	1.000	0.556
**Pulmonary hypertension (PH)**									
Documented presence, n/N (%)	7/10 (70)	2/9 (22)	0.375	5/9 (56)	4/7 (57)	1.000	0.650	0.302	0.385
eRVSP (in PH patients), mmHg	47.1 ± 8.6	36.2 ± 10.1	0.197	60.8 ± 14.7	46.8 ± 16.4	0.125	0.104	0.248	0.805
PH Severity, n/N (%)			0.198			0.149	0.500	0.471	0.808
- No PH	3/10 (30)	7/9 (78)		4/9 (44)	3/7 (43)				
- Mild	4/10 (40)	1/9 (11)		1/9 (11)	1/7 (14)				
- Moderate	3/10 (30)	1/9 (11)		3/9 (33)	3/7 (43)				
- Severe	0/0 (0)	0/9 (0)		1/9 (11)	0/7 (0)				

Data are mean ± SD or n (%). Within-group P: paired pre- vs post-procedural comparison (Wilcoxon signed-rank test for continuous variables and for the ordinal pulmonary hypertension severity score; exact McNemar test for paired binary variables).

Between-group P (Pre, Post): BT-MAT vs historical control — Mann-Whitney U test for continuous variables and Fisher exact test for categorical variables, computed on the patients shown in each cell at the corresponding time point. Change: between-group comparison of the within-patient pre→post change (post minus pre) using the Mann-Whitney U test among paired patients, applied identically to continuous, ordinal severity, and binary variables.

Echocardiographic continuous values for the historical control group were obtained from echocardiography reports. eRVSP, RV S′, TAPSE, and FAC were available only in patients in whom they were measured.

Abbreviations: BT-MAT, bolus thrombolysis plus manual aspiration thrombectomy; eRVSP, estimated right ventricular systolic pressure; FAC, fractional area change; PH, pulmonary hypertension; RV, right ventricular; TAPSE, tricuspid annular plane systolic excursion.

Supplementary Table S3. Outcomes

Outcome	BT-MAT group (n = 11)	Historical control group (n = 12)	p-value
Clinical efficacy			
Clinical success, n (%)*	11 (100)	10 (83)	0.478
Technical success, n (%)	11 (100)	NA	NA
Complications, n (%)†			
Overall complications	0 (0)	2 (17)	0.478
Bloody sputum with pneumonia	0 (0)	1 (8)	
Hematuria‡	0 (0)	1 (8)	
Hospital stay, days			
ICU care	3.0 [2.5–4.5]; 3.7 ± 3.0	0.0 [0.0–5.5]; 3.4 ± 5.0	0.389
GW care	7.0 [4.5–8.5]; 7.9 ± 5.4	10.0 [6.0–19.0]; 11.5 ± 8.7	0.267
Total hospital stay	10.0 [6.5–15.0]; 11.6 ± 7.6	11.5 [7.8–20.5]; 14.9 ± 11.2	0.459

Note. Categorical variables are presented as number (%). Hospital stay is presented as median [interquartile range]; mean ± standard deviation. P-values were calculated using the Mann-Whitney U test or Fisher's exact test as appropriate.

* Absolute risk difference for clinical success: 16.7 percentage points (exact 95% CI, −14.8 to 48.4).

† Absolute risk difference for overall complications: −16.7 percentage points (exact 95% CI, −48.4 to 14.8).

‡ Hematuria was transient and classified as minor bleeding.

Abbreviations: BT-MAT, bolus thrombolysis plus manual aspiration thrombectomy; GW, general ward; ICU, intensive care unit.
